# Complete mitochondrial genome of the clearwing moth *Synanthedon namdoelegans* Kim, Kim and Choi, 2025 (Lepidoptera: Sesiidae)

**DOI:** 10.1080/23802359.2025.2609347

**Published:** 2026-01-01

**Authors:** Seung Hyun Lee, Woo Jin Kim, Jeong Sun Park, Jee-Young Pyo, Iksoo Kim

**Affiliations:** a Department of Applied Biology, College of Agriculture & Life Sciences, Chonnam National University, Gwangju, Republic of Korea; b Department of Forest-Bio, Jeollanam-do Forest Research Institute, Naju-si, Republic of Korea; c Department of Plant Protection and Quarantine, Chonnam National University, Gwangju, Republic of Korea; d Department of Agricultural Biology, National Academy of Agricultural Science, Rural Development Administration, Wanju, Republic of Korea

**Keywords:** Synanthedonini, new species, gene arrangement, South Korea

## Abstract

The clearwing moth *Synanthedon namdoelegans* Kim, Kim and Choi, 2025 (Lepidoptera: Sesiidae) was newly discovered in South Korea in 2024. We sequenced 15,578 bp-long complete mitochondrial genome of the species. It contained the *trnQ–trnS2–trnM–trnI* arrangement at the A + T-rich region and *ND2* junction (the underline indicates counterclockwise direction) unique to tribe Synanthedonini. Phylogenetic analyses of 18 Cossoidea species supported the monophyly of each Synanthedonini and *Synanthedon*, and revealed a sister relationship between S. *namdoelegans* and *Synanthedon bicingulata* (Staudinger 1887). These data will be useful in taxonomic, molecular ecological, and pest management research.

## Introduction

The genus *Synanthedon* Hübner, 1819 (Lepidoptera: Sesiidae) comprises 234 species and nine subspecies (Pühringer and Kallies 2024), with eight species in South Korea (Arita et al. 2004). In 2023 and 2024, we performed field surveys in the southern part of South Korea assessing the seasonal abundance of *Synanthedon bicingulata* (Staudinger 1887), which causes significant damage to urban trees, especially the cherry trees commonly found in city forests and parks. During this survey, a new species of *Synanthedon* was discovered on a cherry tree (Kim et al. 2025). Morphological analysis, along with the DNA barcoding sequences from four individuals, supported the novelty of this species, which was named *Synanthedon namdoelegans* Kim, Kim and Choi, 2025 (Kim et al. 2025).

To supplement this finding, extended molecular data could be essential. Notably, mitochondrial genome (mitogenome) sequences are available for only five *Synanthedon* species. Thus, additional species’ genomes will be required for the further exploration of evolutionary and phylogenetic relationships within this genus.

In this study, we sequenced the complete mitogenome of *S. namdoelegans* and compared it with the mitogenomes of other Cossoidea superfamily members to understand its baseline genomic features and phylogenetic relationships.

## Materials and methods

**Figure 1.**
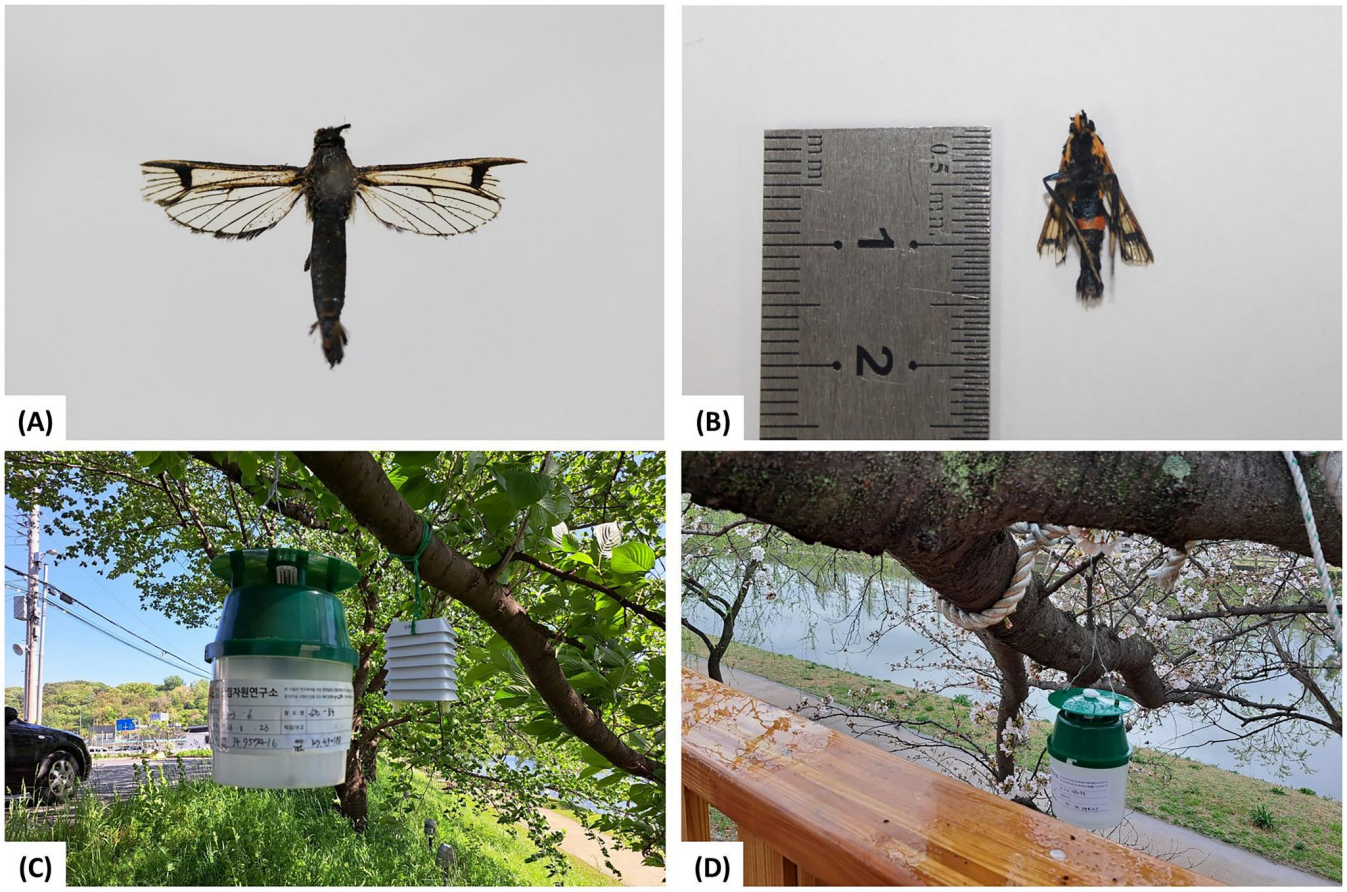
Images of *Synanthedon namdoelegans* Kim, Kim and Choi, 2025 and the pheromone traps used in the survey: (A) dorsal and (B) ventral views of an *S. namdoelegans* adult and (C, D) pheromone traps installed on a cherry tree (*Prunus* × *yedoensis* Matsumura 1901). The photos were taken by the authors, Jee-Young Pyo and Woo Jin Kim.

An adult male *S. namdoelegans* individual collected in Suncheon-si, Jeollanam-do, South Korea (34°57′37.8ʺ N, 127°29′20.8ʺ E) was used for molecular analysis. The specimen was captured using an *S. bicingulata*-specific pheromone lure in a funnel-trap (170 × 250 mm; Green Agrotech, Gyeongsan, South Korea) installed on the branches of a cherry tree (*Prunus* ×* yedoensis* Matsumura 1901) on 26 April 2023 (Figure 1). Species identification was made by one of the authors (Iksoo Kim) based on morphological characteristics, described in Kim et al. (2025) and the DNA barcode sequences reported in Kim et al. (2025).

Genomic DNA was extracted from two hind legs using the Wizard™ Genomic DNA Purification Kit (Promega, Madison, WI). A specimen and leftover DNA were deposited at the Chonnam National University, Gwangju, South Korea (https://www.jnu.ac.kr/, Iksoo Kim, ikkim81@chonnam.ac.kr) under the voucher number CNU17887. Using the genomic DNA as a template, three long-overlapping fragments (*COX1*–*ND4*, *ND5*–*16S rRNA*, and *16S rRNA*–*COX1*) were amplified using primers described in a previous study (Kim et al. 2012) along with a few primers newly designed for this study (Table S1). Subsequently, 26 short overlapping fragments were amplified using the long fragments as templates (Figure S1). Bidirectional sequencing was conducted using the Sanger method, and the SeqMan tool from the DNASTAR software package (SeqMan NGen^®^ v13.0, DNASTAR, Madison, WI) was used to assemble the 26 overlapping fragments into a complete mitogenome. Individual genes and the A + T-rich region were annotated by aligning their sequences with homologous sequences from known full-length lepidopteran mitogenomes using MAFFT v7 (Katoh et al. 2002). The nucleotide sequences of protein-coding genes (PCGs) were aligned based on codons using RevTrans v2.0 (Wernersson and Pedersen 2003), and tRNA genes were identified using tRNAscan-SE v2.0 (Lowe and Eddy 1997).

The baseline mitogenome information of 17 mitogenomes from species in the Cossoidea superfamily was downloaded from GenBank (as of 10 October 2025). Using these mitogenomes, along with that of *S. namdoelegans*, phylogenetic analyses were conducted based on 13 PCGs and two rRNAs (13,732 bp, including gaps). *Leguminivora glycinivorella* (Matsumura 1898) (unpublished) and *Grapholita funebrana* (Treitschke, 1835) (unpublished), belonging to the Tortricoidea superfamily, were used as the outgroups. Phylogenetic analyses were performed using MrBayes for Bayesian inference tree (Ronquist et al. 2012) and IQ-TREE for maximum-likelihood tree (Nguyen et al. 2015), implemented in PhyloSuite v1.2.3 (Xiang et al. 2023). The optimal partitioning scheme (four for MrBayes and nine partitions for IQ-TREE) and corresponding substitution models (Table S2) were determined using PartitionFinder 2 with the greedy algorithm (Lanfear et al. 2017). The genetic distance within the Synanthedonini tribe, to which current *S. namdoelegans* is included, was calculated using all 37 genes to know the sequence divergence of *S. namdoelegans* from other *Synanthedon* species. The calculation was performed using unrooted pairwise distances estimated with PAUP ver. 4.01b10 (Swofford 2002).

## Results

**Figure 2.**
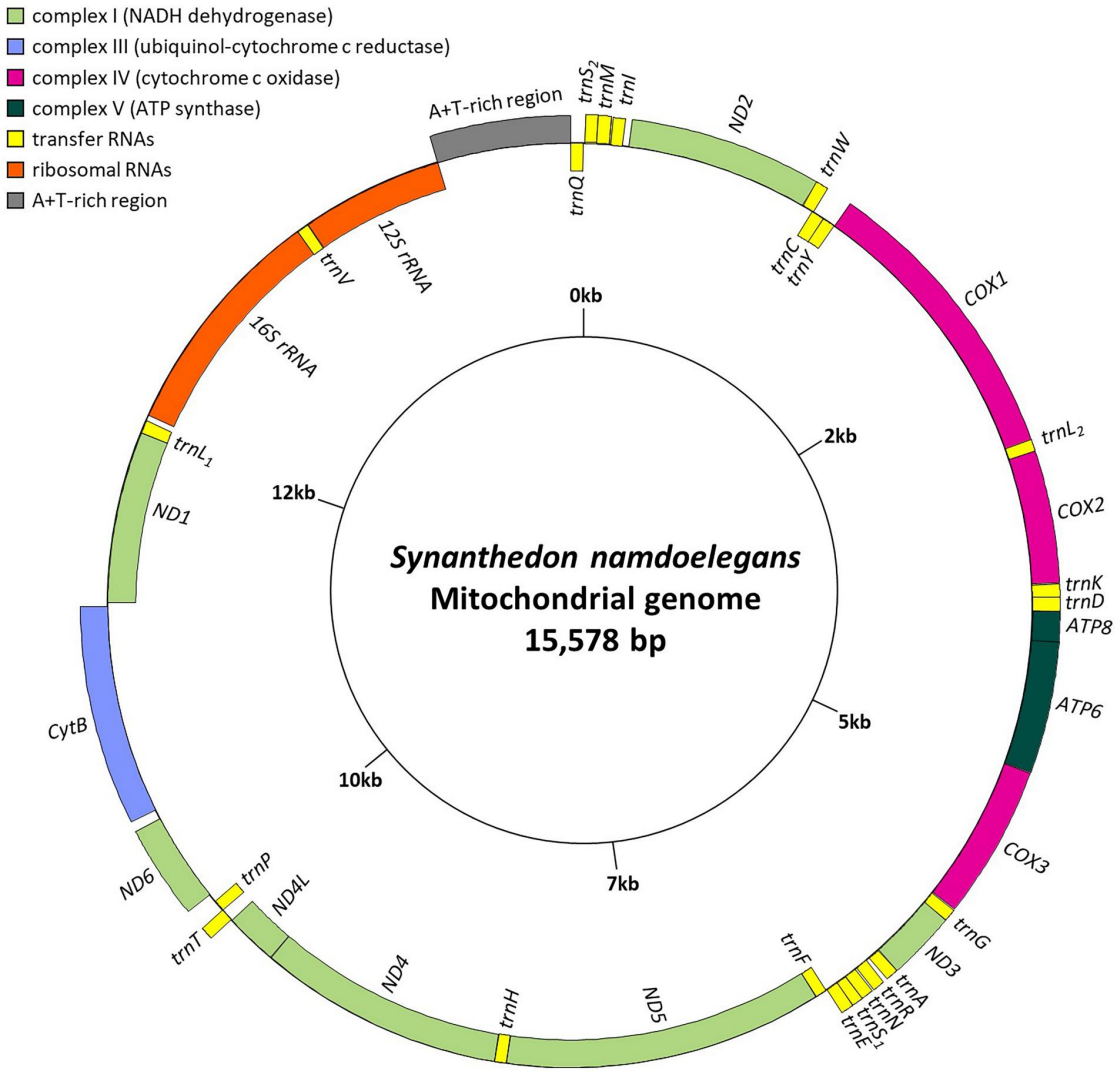
Circular map of the mitochondrial genome of *Synanthedon namdoelegans* obtained using the GenomeVx tool (http://wolfe.ucd.ie/GenomeVx/). The tRNA abbreviations follow the IUPAC-IUB one-letter code, with *trnL_1_*, *trnL_2_*, *trnS_1_*, and *trnS_2_* denoting *tRNA^Leu^*(CUN), *tRNA^Leu^*(UUR), *tRNA^Ser^*(AGN), and *tRNA^Ser^*(UCN), respectively. Genes with names outside the circular map are transcribed in the clockwise direction (excluding the A + T-rich region), while those with names inside the map are transcribed in the counterclockwise direction.

The 15,578-bp long complete mitogenome of *S. namdoelegans* contained two rRNAs, 22 tRNAs, 13 PCGs, and an A + T-rich region (Figure 2). Eleven PCGs started with typical ATN codons, whereas *COX1* and *ATP8* used atypical start codons, CGA and TTG, respectively (Table 1). The PCGs *COX1*, *ND5*, and *ND4* had the incomplete stop codon T, whereas the remaining PCGs terminated with TAA or TAG (Table 1). The *S. namdoelegans* mitogenome had a rearranged gene block (*trnQ*–*trnS_2_*–*trnM*–*trnI* (the underline indicates a counterclockwise transcriptional direction)) between the A + T-rich region and *ND2*, which differs from the typical gene arrangement seen in Lepidoptera, *trnM*–*trnI*–*trnQ* (Figure S2). The A/T content was highest in the A + T-rich region (91.29%), followed by the *12S rRNA* gene (85.82%), *16S rRNA* gene (85.10%), 22 tRNA genes (83.46%), whole genome (79.18%), and 13 PCGs (76.50%) (Table S3).

**Table 1.** Summary of *Synanthedon namdoelegans*’ mitochondrial genome.

Gene	Nucleotide range	Size (bp)	Anticodon	Codon		O/S
				Start	Stop	
*trnQ*	1–68	68	TTG	–	–	
*trnS_2_*	78–139	62	TGA	–	–	−9
*trnM*	142–208	67	CAT	–	–	−2
*trnI*	218–281	64	GAT	–	–	−9
*ND2*	312–1304	993	–	ATA	TAA	−30
*trnW*	1306–1372	67	TCA	–	–	−1
*trnC*	1365–1436	72	GCA	–	–	+8
*trnY*	1437–1503	67	GTA	–	–	
*COX1*	1507–3037	1531	–	CGA	T-tRNA	−3
*trnL_2_*	3038–3101	64	TAA	–	–	
*COX2*	3102–3782	681	–	ATA	TAA	
*trnK*	3784–3854	71	CTT	–	–	−1
*trnD*	3854–3925	72	GTC	–	–	+1
*ATP8*	3926–4087	162	–	TTG	TAA	
*ATP6*	4081–4758	678	–	ATG	TAA	+7
*COX3*	4763–5557	795	–	ATG	TAA	−4
*trnG*	5562–5625	64	TCC	–	–	−4
*ND3*	5626–5979	354	–	ATT	TAA	
*trnA*	5981–6046	66	TGC	–	–	−1
*trnR*	6068–6133	66	TCG	–	–	−21
*trnN*	6141–6205	65	GTT	–	–	−7
*trnS_1_*	6208–6266	59	GCT	–	–	−2
*trnE*	6267–6332	66	TTC	–	–	
*trnF*	6335–6398	64	GAA	–	–	−2
*ND5*	6399–8133	1735	–	ATT	T-tRNA	
*trnH*	8134–8199	66	GTG	–	–	
*ND4*	8200–9538	1339	–	ATG	T-tRNA	
*ND4L*	9539–9823	285	–	ATG	TAG	
*trnT*	9829–9897	69	TGT	–	–	−5
*trnP*	9897–9962	66	TGG	–	–	+1
*ND6*	10,009–10,494	486	–	ATA	TAA	−46
*CytB*	10,525–11,676	1152	–	ATG	TAA	−30
*ND1*	11,696–12,631	936	–	ATG	TAG	−19
*trnL_1_*	12,633–12,700	68	TAG	–	–	−1
*16S rRNA*	12,734–14,015	1282	–	–	–	−33
*trnV*	14,016–14,079	64	TAC	–	–	
*12S rRNA*	14,080–14,855	776	–	–	–	
A + T-rich region	14,856–15,578	723	–	–	–	

Genes with non-underlined names (excluding the A + T-rich region) are transcribed in the clockwise direction, whereas those with underlined names are transcribed in the counterclockwise direction. The tRNA abbreviations follow the IUPAC-IUB one-letter code, with *trnL_1_*, *trnL_2_*, *trnS_1_*, and *trnS_2_* denoting *tRNA^Leu^*(CUN), *tRNA^Leu^*(UUR), *tRNA^Ser^*(AGN), and *tRNA^Ser^*(UCN), respectively. The O/S column quantifies the number of overlapping (+)/intergenic spacer (−) sequences.

**Figure 3.**
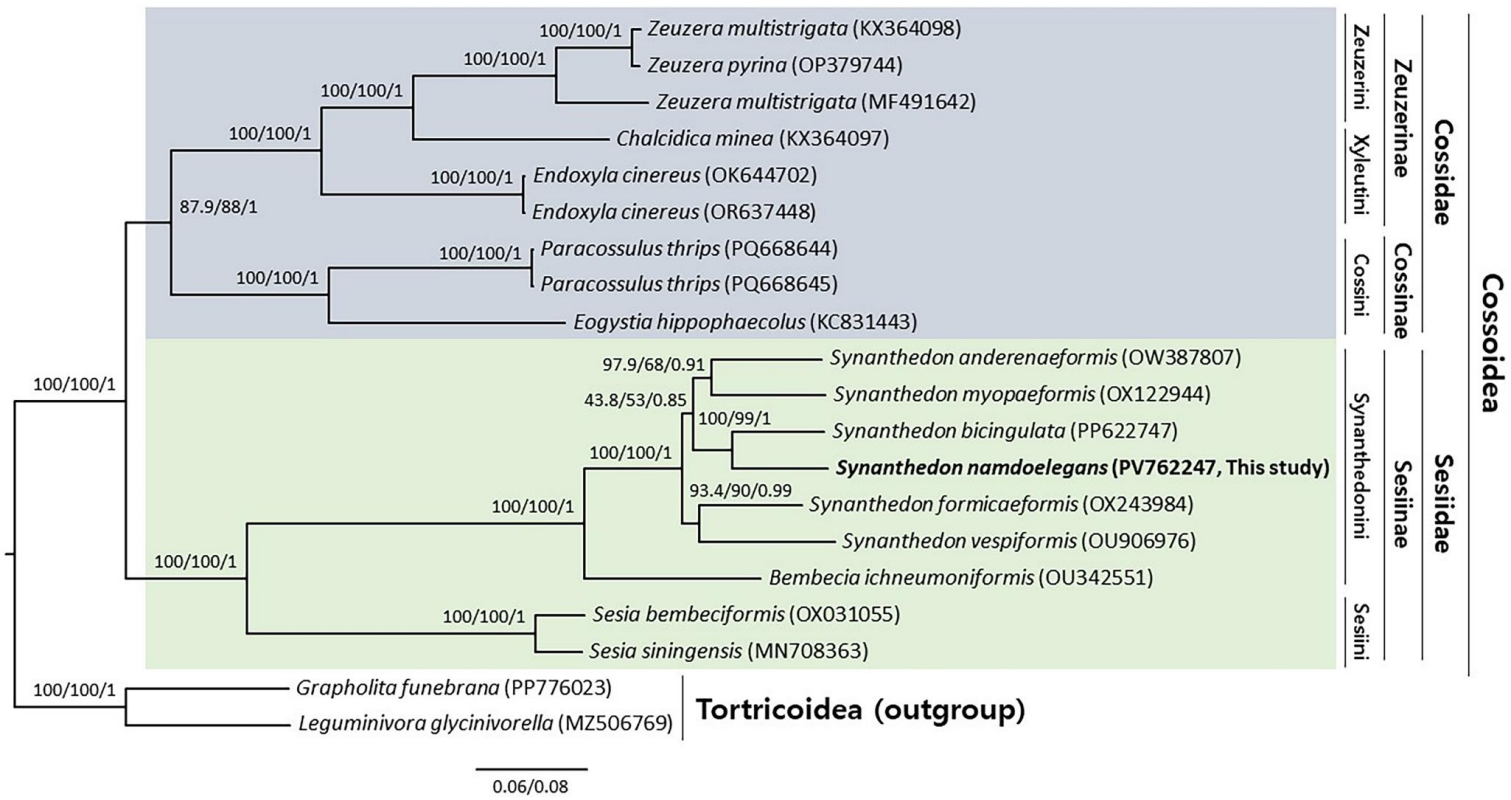
Phylogeny of 18 mitochondrial genomes from members of the families Sesiidae and Cossidae, which constitute the Cossoidea superfamily, including *Synanthedon namdoelegans* (in bold). The tree was derived using maximum-likelihood and Bayesian inference methods. The numbers at each node are Shimodaira–Hasegawa-like approximate likelihood ratio test (SH-aLRT) support values, ultrafast bootstrap (UFBoot) support values, and Bayesian posterior probabilities (BPPs), respectively (SH-aLRT/UFBoot/BPP). The scale bar indicates the number of substitutions per site. *Grapholita funebrana* and *Leguminivora glycinivorella*, belonging to the Tortricoidea superfamily, were used as the outgroups. The analyzed sequences include *Eogystia hippophaecolus* (KC831443; Gong et al. 2014), *Paracossulus thrips* (PQ668644 and PQ668645; Jordán et al. 2025), *Chalcidica minea* (KX364097; Li et al. 2018), *Endoxyla cinereus* (OK644702; unpublished), *Endoxyla cinereus* (OR637448; Cameron 2023), *Zeuzera multistrigata* (MF491642; Kim et al. 2017), *Zeuzera multistrigata* (KX364098; Li et al. 2018), *Zeuzera pyrina* (OP379744; Cheng et al. 2022), *Sesia bembeciformis* (OX031055; Boyes et al. 2023b), *Sesia siningensis* (MN708363; Yan et al. 2019), *Bembecia ichneumoniformis* (OU342551; Boyes et al. 2023a), *Synanthedon andrenaeformis* (OW387807; Boyes et al. 2024), *Synanthedon bicingulata* (PP622747; Kim et al. 2024), *Synanthedon formicaeformis* (OX243984; Langdon and Fagan 2023), *Synanthedon myopaeformis* (OX122944; Langdon and Holland 2024), *Synanthedon namdoelegans* (PV762247; this study), *Synanthedon vespiformis* (OU906976; Boyes et al. 2022), *Grapholita funebrana* (PP776023; unpublished), and *Leguminivora glycinivorella* (MZ506769; unpublished).

The genetic distance of *S. namdoelegans* to other *Synanthedon* species ranged from 7.54% (1115 bp, *S. bicingulata*) to 9.57% (1416 bp, *Synanthedon myopaeformis* (Borkhausen, 1789) and *Synanthedon vespiformis* (Linnaeus 1761)) in the range from 8.79% (1301 bp) to 10.26% (1518 bp) within *Synanthedon* (Table S4). Phylogenetic analysis showed that the *Synanthedon* genus, Synanthedonini tribe, and Sesiinae subfamily, to which *S. namdoelegans* belongs, are monophyletic groups with the highest nodal supports (Shimodaira–Hasegawa-like approximate likelihood ratio test (SH-aLRT) = 100, ultrafast bootstrap (UFBoot) = 100, Bayesian posterior probabilities (BPPs) = 1) (Figure 3). *S. namdoelegans* was placed as the sister taxon to the *S. bicingulata*, with a relatively high nodal support (SH-aLRT = 100, UFBoot = 99, BPP = 1) (Figure 3).

## Discussion and conclusions

In this study, we sequenced the complete mitogenome of *S. namdoelegans*, a newly discovered *Synanthedon* species from South Korea. The relative A/T contents among the whole mitogenome, genes, and the A + T-rich region in *S. namdoelegans* were largely consistent with those of other Cossoidea species, though a few species differed in the relative A/T contents of their *12S rRNA* gene, *16S rRNA* gene, and 22 tRNA genes (Table S3), indicating that relative A/T content is a conserved mitogenome characteristic in the Cossoidea superfamily. Although the start codons for *COX1* (CGA) and *ATP8* (TTG) are not canonical, the CGA codon for *COX1* is a highly conserved feature in Lepidoptera, and TTG is common in insect PCGs, including those of lepidopterans (Kim et al. 2009; Zhao et al. 2011; Jeong et al. 2022). The gene rearrangement identified in *S. namdoelegans*’ mitogenome (*trnQ*–*trnS_2_*–*trnM*–*trnI*) is seen in all species in the Synanthedonini tribe at the same junction, but differs from the typical *trnM*–*trnI*–*trnQ* arrangement found in Lepidoptera, with a few exceptions (Figure S2; Cao et al. 2012; Timmermans et al. 2014). This consistency suggests it is a synapomorphic trait for the tribe. However, additional research is necessary to validate this hypothesis. The observed rearrangement may have resulted from the tandem duplication–random loss model, which can generate new arrangements (Moritz et al. 1987; Cameron 2014).

From the taxonomic perspective, our findings are consistent with the previous study (Kim et al. 2025), which demonstrated the distinctiveness of *S. namdoelegans* based on both morphological traits and mitochondrial gene sequences (the partial sequence of *COX1* and *ND1*). In that study, *S. namdoelegans* was closely related to *Synanthedon soffneri* Špatenka, 1983, *Synanthedon bastak* Gorbunov & Koshkin, 2023, and *Synanthedon velox* (Fixsen, 1887), in some morphological characters, but was distinguished by differences in the forewing’s black band, the hindwing’s discoidal vein spot, and male genitalia (Kim et al. 2025). The present mitogenome analysis further supports this conclusion, showing that *S. namdoelegans* is robustly separated from its congeners in mitogenome sequences (Table S4). As *S. namdoelegans* is a newly discovered species (Kim et al. 2025), the addition of its mitogenome sequence could facilitate a variety of applications, including species identification, molecular ecology, pest management, and phylogenetic studies. In particular, the robustness of the sister relationship between *S. namdoelegans* and *S. bicingulata* could be further tested by incorporating mitogenome sequences from other *Synanthedon* species and nuclear DNA fragments into future phylogenetic analyses.

## Supplementary Material

Figure S1_PCR gel plot.jpg

Table S1_List of primers.docx

Figure S2_Linear arrangement.jpg

Table S2_Results of PartitionFinder_revised.docx

Table S4_All gene pairwise comparison.docx

Table S3_Characteristics of Cossoidea_revised.docx

## Data Availability

The genome sequence data that support the findings of this study are openly available in GenBank of NCBI at https://www.ncbi.nlm.nih.gov under the accession no. PV762247. The chromatography files are available in Mendeley Data (https://doi.org/10.17632/jmwsgx59m9.1).
