# Supplementary figures and images for: Complete mitochondrial genome of the clearwing moth Synanthedon namdoelegans Kim, Kim and Choi, 2025 (Lepidoptera: Sesiidae)
Source: Mitochondrial DNA B Resour. 2026 Jan 1;11(1):195–200. doi: 10.1080/23802359.2025.2609347 (PMC12777775; doi:10.1080/23802359.2025.2609347)

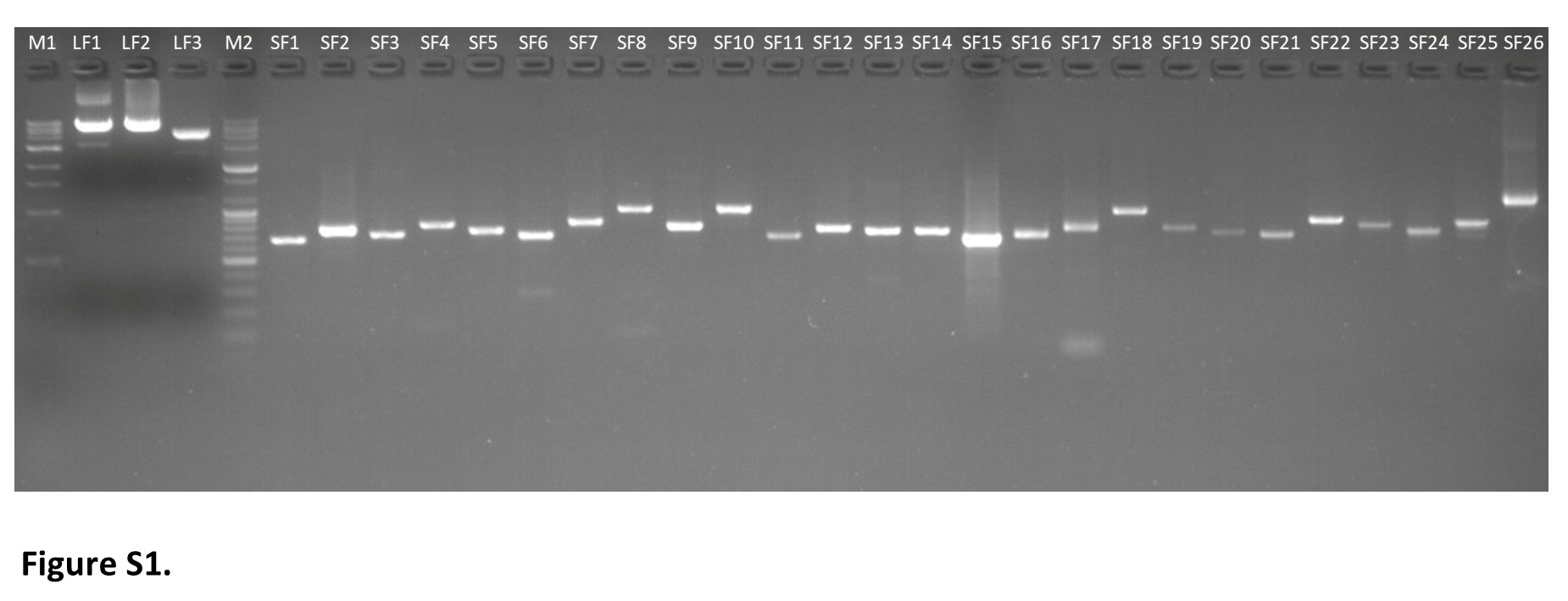

Supplement: Figure S1_PCR gel plot.jpg [file TMDN_A_2609347_SM7664.jpg]

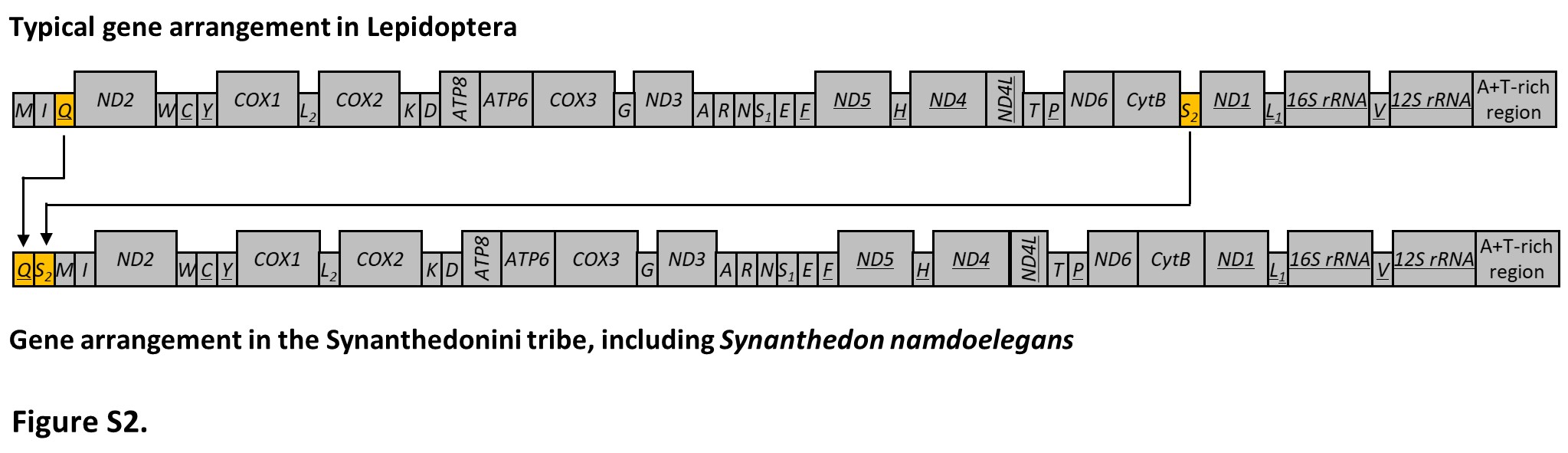

Supplement: Figure S2_Linear arrangement.jpg [file TMDN_A_2609347_SM7662.jpg]
