# Supplementary material for: Complete mitochondrial genome of the clearwing moth Synanthedon namdoelegans Kim, Kim and Choi, 2025 (Lepidoptera: Sesiidae)
Source: Mitochondrial DNA B Resour. 2026 Jan 1;11(1):195–200. doi: 10.1080/23802359.2025.2609347 (PMC12777775; doi:10.1080/23802359.2025.2609347)
Supplement: Table S1_List of primers.docx [file TMDN_A_2609347_SM7663.docx]

**Table S1.** List of primers used to amplify and sequence the mitochondrial genome of *Synanthedon namdoelegans*.

| Fragment name | Primer name^a^ | Direction^b^ | Sequence (5’–3’) | Nucleotide range^c^ |
| --- | --- | --- | --- | --- |
| *Long fragments* |  |  |  |  |
| LF1 | Synanthedon-LF1-F1 | F | AAGTCTTTTAATTCGTGCTG | 1596–1615 |
| LF1 | Synanthedon-LF1-R1 | R | AATTAATAGTTTAGTGGGWTGATC | 8409–8432 |
| LF2 | Lep-ND5-F2 | F | CGAATATCTTGAATATCATTTATTA | 8032–8056 |
| LF2 | Lep-lrRNA-R2 | R | GTATCTTGTGTATCAGAGTTTA | 13849–13870 |
| LF3 | Lep-lrRNA-F1 | F | TGTAAGATTTTAATGATCGAACAGAT | 12851–12876 |
| LF3 | Lep-COI-R1 | R | CTTCAGGATGACCAAAAAATC | 2204–2224 |
| *Short fragments* |  |  |  |  |
| SF1 | LF03-S05-F2 | F | TWAAAGGATTATTCTGATAG | 232–251 |
| SF1 | LF03-S05-R2 | R | CAWCCTAAATTATTAATWGAWGA | 819–841 |
| SF2 | LF03-S06-F2 | F | ATTRTWGAAGGWTTATCWTG | 636–655 |
| SF2 | LF03-S06-R1 | R | GATATAAAATTGCAAATTTTAAG | 1392–1414 |
| SF3 | COIF | F | CTTAAAATTTGCAATTTTATATC | 1392–1414 |
| SF3 | Lep-COI-R1 | R | CTTCAGGATGACCAAAAAATC | 2204–2224 |
| SF4 | LF01-S01-F2 | F | TTACAACAATTATTAATATACG | 1991–2012 |
| SF4 | LF01-S01-R2 | R | GTCGAGGTATTCCTGCTA | 2795–2812 |
| SF5 | LF01-S02-F2 | F | ACWGTAGGAGGATTAACAGG | 2542–2561 |
| SF5 | LF01-S02-R2 | R | GTTCAAATTAATTCAATTATTTG | 3276–3298 |
| SF6 | LF01-S03-F2 | F | TAGAAATGGCAACWTGATC | 3097–3115 |
| SF6 | LF01-S03-R1 | R | CTTGCTTTCAGTCATCTAAT | 3785–3804 |
| SF7 | LF01-S04-F1 | F | CAGGTCGWTTAAATCAAAC | 3628–3646 |
| SF7 | LF01-S04-R2 | R | GTTCCTTGDGGAATTATATG | 4467–4486 |
| SF8 | LF01-S05-F1 | F | TTATTTTCAATTTTTGATCC | 4093–4112 |
| SF8 | LF01-S05-R2 | R | CCAATTTCAATATTAGGDGATA | 5088–5109 |
| SF9 | LF01-S06-F2 | F | GTWGATTATAGHCCWTGACC | 4802–4821 |
| SF9 | LF01-S06-R2 | R | GATTGGAAGTCAAATATACT | 5579–5598 |
| SF10 | LF01-S07-F1 | F | ATTTCTTTTAATTTGCTTAATTCG | 5410–5433 |
| SF10 | LF01-S07-R1 | R | CAATTTTATCATTAACAGTGA | 6296–6316 |
| SF11 | LF01-S08-F2 | F | GAAATCAAAATATATTAAATTG | 5948–5969 |
| SF11 | LF01-S08-R1 | R | TGATTTATACCTARWTTATCWAC | 6617–6639 |
| SF12 | LF01-S09-F1 | F | AWAHTTCTCTTCAACCYAWATC | 6540–6561 |
| SF12 | LF01-S09-R2 | R | GCTTTATCWACTTTAAGWCA | 7295–7314 |
| SF13 | LF01-S10-F1 | F | TCYTTWGAATAAAAYCCAG | 7043–7061 |
| SF13 | LF01-S10-R1 | R | GATGGDTTAGGDTTAGTTTCTT | 7764–7785 |
| SF14 | LF01-S11-F1 | F | AAAAAATATAATTTCAWCTHCC | 7623–7644 |
| SF14 | LF01-S11-R2 | R | GAGCTGGDTATAGATTATAT | 8348–8367 |
| SF15 | LF01-S12-F2 | F | ATATTTTTGAYHCCACAAATC | 8154–8174 |
| SF15 | Synanthedon-SF15-R1 | R | ATTAGATTAAAGTGTTTATGTC | 8740–8761 |
| SF16 | LF02-S01-F1 | F | TTATAATACCHCCAATWAC | 8668–8686 |
| SF16 | LF02-S01-R1 | R | GGTTTAATTTTATTAAGAATTTG | 9239–9261 |
| SF17 | LF02-S02-F1 | F | ATATTAAAGTAGGAATTAAWC | 9165–9185 |
| SF17 | LF02-S02-R2 | R | TAATTTTGGAGATTATWGAT | 9918–9937 |
| SF18 | LF02-S03-F1 | F | CCTAAAGCHCCYTCACAAAC | 9610–9629 |
| SF18 | LF02-S03-R2 | R | GGTAAATCAATTAAWGATCYAT | 10580–10601 |
| SF19 | LF02-S04-F2 | F | TNTCAAGAATTGCHTCWAATG | 10169–10189 |
| SF19 | LF02-S04-R1 | R | GATATTTGTCCYCAAGGTA | 10934–10952 |
| SF20 | LF02-S05-F2 | F | TATHTHCATATTGGACGAGG | 10819–10838 |
| SF20 | LF02-S05-R2 | R | CCAATTCAWGTTAATAAAAT | 11533–11552 |
| SF21 | LF02-S06-F2 | F | ACHCCHRTTCATATTCAACC | 11326–11345 |
| SF21 | LF02-S06-R2 | R | GCTGAAACTAATCGAACTC | 12022–12040 |
| SF22 | LF02-S07-F2 | F | AAAGCAAATCCCCCTCTTC | 11946–11964 |
| SF22 | LF02-S07-R2 | R | CTGAGTTCAAACCGGTGTRA | 12827–12846 |
| SF23 | LF02-S08-F1 | F | TCTAATAAAGTTAAAAAAGC | 12540–12559 |
| SF23 | LF02-S08-R2 | R | CACTTGTTTATCAAAAACATGTC | 13357–13379 |
| SF24 | LF03-S02-F1 | F | ATTATGCTACCTTTGTACAGTC | 13273–13294 |
| SF24 | LF03-S02-R1 | R | GTATTTCATTTACATTGAAAAGA | 14038–14060 |
| SF25 | LF03-S03-F3 | F | CTCTGATACACAAGATAC | 13853–13870 |
| SF25 | LF03-S03-R3 | R | CCAGCAGTTGCGGTTAAAC | 14714–14732 |
| SF26 | LF03-S04-F2 | F | ATTTTTTATTAAATTAAAATTTCAC | 14618–14642 |
| SF26 | Synanthedon-SF26-R1 | R | ATACTTTATTTACCCTATCAG | 245–265 |

^a^Most primers were adapted from Kim et al. (2012), but Synanthedon-LF1-F1, Synanthedon-LF1-R1, Synanthedon-SF15-R1, and Synanthedon-SF26-R1 were newly designed in this study using available *Synanthedon* mitochondrial genome sequences. ^b^F and R indicate forward and reverse primers, respectively. ^c^Nucleotide ranges are based on *Synanthedon namdoelegans*’ mitochondrial genome.
