# Supplementary material for: Complete mitochondrial genome of the clearwing moth Synanthedon namdoelegans Kim, Kim and Choi, 2025 (Lepidoptera: Sesiidae)
Source: Mitochondrial DNA B Resour. 2026 Jan 1;11(1):195–200. doi: 10.1080/23802359.2025.2609347 (PMC12777775; doi:10.1080/23802359.2025.2609347)
Supplement: Table S2_Results of PartitionFinder_revised.docx [file TMDN_A_2609347_SM7661.docx]

| Schemes (AIC) | Model | Partition |
| --- | --- | --- |
| Subset 1 | GTR + I + G | *12S rRNA* |
| Subset 2 | GTR + I + G | *16S rRNA* |
| Subset 3 | GTR + I + G | *ATP6*, *ND3* |
| Subset 4 | GTR + I + G | *ND6*, *ATP8* |
| Subset 5 | GTR + I + G | *COX1*, *COX2*, *COX3* |
| Subset 6 | GTR + I + G | *CytB* |
| Subset 7 | GTR + I + G | *ND1* |
| Subset 8 | GTR + I + G | *ND2* |
| Subset 9 | GTR + I + G | *ND4L*, *ND5*, *ND4* |
| Schemes (BIC) | Model | Partition |
| Subset 1 | GTR + I + G | *12S rRNA*, *16S rRNA* |
| Subset 2 | GTR + I + G | *ND2*, *ATP6*, *ND3*, *ATP8*, *ND6* |
| Subset 3 | GTR + I + G | *CytB*, *COX1*, *COX3*, *COX2* |
| Subset 4 | GTR + I + G | *ND1*, *ND4L*, *ND5*, *ND4* |

**Table S2.** Optimal substitution models corresponding to each partition, selected using PartitionFinder 2 based on the Akaike information criterion (AIC) or the Bayesian information criterion (BIC).
