# Supplementary material for: Complete mitochondrial genome of the clearwing moth Synanthedon namdoelegans Kim, Kim and Choi, 2025 (Lepidoptera: Sesiidae)
Source: Mitochondrial DNA B Resour. 2026 Jan 1;11(1):195–200. doi: 10.1080/23802359.2025.2609347 (PMC12777775; doi:10.1080/23802359.2025.2609347)
Supplement: Table S4_All gene pairwise comparison.docx [file TMDN_A_2609347_SM7660.docx]

**Table S4.** Pairwise comparisons among Synanthedonini species based on 37 genes (13 PCGs, 2 rRNAs and 22 tRNAs).

| No. | Species (GenBank No.) | 1 | 2 | 3 | 4 | 5 | 6 | 7 |
| --- | --- | --- | --- | --- | --- | --- | --- | --- |
| 1 | *Bembecia ichneumoniformis* (OU342551) | - | 11.78 | 11.92 | 12.17 | 11.87 | 12.11 | 12.47 |
| 2 | *Synanthedon anderenaeformis* (OW387807) | 1,743 | - | 9.04 | 9.22 | 8.79 | 9.36 | 10.09 |
| 3 | *Synanthedon bicingulata* (PP622747) | 1,764 | 1,338 | - | 9.38 | 9.06 | 7.54 | 10.08 |
| 4 | *Synanthedon formicaeformis* (OX243984) | 1,800 | 1,364 | 1,387 | - | 9.69 | 8.96 | 9.51 |
| 5 | *Synanthedon myopaeformis* (OX122944) | 1,756 | 1,301 | 1,340 | 1,433 | - | 9.57 | 10.26 |
| 6 | ***Synanthedon namdoelegans* (PV762247, This study)** | 1,792 | 1,384 | 1,115 | 1,325 | 1,416 | - | 9.57 |
| 7 | *Synanthedon vespiformis* (OU906976) | 1,845 | 1,493 | 1,492 | 1,407 | 1,518 | 1,416 | - |

The numbers above the diagonal are the mean distance values; the numbers below the diagonal are the absolute distance values.
