# Supplementary material for: Complete mitochondrial genome of the clearwing moth Synanthedon namdoelegans Kim, Kim and Choi, 2025 (Lepidoptera: Sesiidae)
Source: Mitochondrial DNA B Resour. 2026 Jan 1;11(1):195–200. doi: 10.1080/23802359.2025.2609347 (PMC12777775; doi:10.1080/23802359.2025.2609347)
Supplement: Table S3_Characteristics of Cossoidea_revised.docx [file TMDN_A_2609347_SM7659.docx]

Table S3. Characteristics of mitochondrial genomes of Cossoidea species.

| Taxon | Size  (bp) | A/T (%) | PCG^a^ | | *12S rRNA* | | *16S rRNA* | | tRNA | | A+T-rich region | | GenBank accession number | Reference |
| --- | --- | --- | --- | --- | --- | --- | --- | --- | --- | --- | --- | --- | --- | --- |
|  |  |  | Number of codons^a^ | A/T (%) | Size (bp) | A/T (%) | Size (bp) | A/T (%) | Size (bp) | A/T (%) | Size (bp) | A/T (%) |  |  |
| Cossoidea |  |  |  |  |  |  |  |  |  |  |  |  |  |  |
| Cossidae |  |  |  |  |  |  |  |  |  |  |  |  |  |  |
| Cossinae |  |  |  |  |  |  |  |  |  |  |  |  |  |  |
| Cossini |  |  |  |  |  |  |  |  |  |  |  |  |  |  |
| *Eogystia hippophaecolus* | 15,431 | 78.43 | 3,731 | 76.79 | 779 | 85.49 | 1,364 | 82.40 | 1,488 | 81.05 | 317 | 92.74 | KC831443 | Gong et al. (2014) |
| *Paracossulus thrips* | 15,395 | 77.66 | 3,723 | 75.19 | 776 | 85.18 | 1,336 | 82.04 | 1,481 | 81.09 | 375 | 93.33 | PQ668644 | Jordán et al. (2025) |
| *Paracossulus thrips* | 15,385 | 77.66 | 3,723 | 75.20 | 776 | 85.18 | 1,334 | 82.01 | 1,481 | 81.16 | 375 | 93.60 | PQ668645 | Jordán et al. (2025) |
| Xyleutini |  |  |  |  |  |  |  |  |  |  |  |  |  |  |
| *Chalcidica minea* | 15,490 | 82.09 | 3,718 | 80.52 | 780 | 86.03 | 1,379 | 84.77 | 1,487 | 82.72 | 354^b^ | 95.76 | KX364097 | Li et al. (2018) |
| *Endoxyla cinereus* | 15,285 | 81.61 | 3,717 | 80.26 | 766 | 85.77 | 1,298 | 84.67 | 1,474 | 81.75 | 308 | 95.13 | OK644702 | Unpublished |
| *Endoxyla cinereus* | 15,319 | 81.66 | 3,715 | 80.28 | 782 | 86.06 | 1,393 | 85.21 | 1,474 | 81.75 | 351 | 94.30 | OR637448 | Cameron (2023) |
| Zeuzerini |  |  |  |  |  |  |  |  |  |  |  |  |  |  |
| *Zeuzera multistrigata* | 15,260 | 78.92 | 3,716 | 76.88 | 759 | 84.72 | 1,346 | 84.62 | 1,465 | 80.41 | 374 | 93.85 | MF491642 | Kim et al. (2017) |
| *Zeuzera multistrigata* | 15,320 | 78.87 | 3,722 | 76.90 | 786 | 83.46 | 1,346 | 83.58 | 1,476 | 81.10 | 356^b^ | 93.26 | KX364098 | Li et al. (2018) |
| *Zeuzera pyrina* | 15,324 | 78.89 | 3,692 | 76.76 | 786 | 83.72 | 1,320 | 83.71 | 1,470 | 81.02 | 353 | 93.48 | OP379744^c^ | Cheng et al. (2022) |
| Sesiidae |  |  |  |  |  |  |  |  |  |  |  |  |  |  |
| Sesiinae |  |  |  |  |  |  |  |  |  |  |  |  |  |  |
| Sesiini |  |  |  |  |  |  |  |  |  |  |  |  |  |  |
| *Sesia bembeciformis* | 16,056 | 80.19 | 3,692 | 77.24 | 774 | 85.53 | 1,320 | 83.56 | 1,466 | 81.17 | 1,005 | 97.61 | OX031055^c^ | Boyes and Langdon (2023) |
| *Sesia siningensis* | 15,454 | 79.65 | 3,692 | 77.37 | 773 | 86.03 | 1,322 | 83.36 | 1,462 | 81.12 | 385 | 98.18 | MN708363 | Yan et al. (2020) |
| Synanthedonini |  |  |  |  |  |  |  |  |  |  |  |  |  |  |
| *Bembecia ichneumoniformis* | 15,323 | 78.71 | 3,701 | 76.22 | 766 | 85.77 | 1,285 | 83.66 | 1,441 | 82.10 | 579 | 93.96 | OU342551^c^ | Boyes (2023) |
| *Synanthedon andrenaeformis* | 16,650 | 80.06 | 3,701 | 76.35 | 762 | 85.17 | 1,295 | 82.63 | 1,446 | 82.37 | 1,746 | 96.05 | OW387807^c^ | Boyes and Holland (2024) |
| *Synanthedon bicingulata* | 16,255 | 79.66 | 3,697 | 76.51 | 759 | 84.98 | 1,288 | 85.09 | 1,451 | 82.43 | 759 | 94.07 | PP622747 | Kim et al. (2024) |
| *Synanthedon formicaeformis* | 15,814 | 78.58 | 3,693 | 75.39 | 761 | 85.28 | 1,286 | 82.66 | 1,452 | 82.85 | 1,039 | 94.71 | OX243984^c^ | Langdon and Fagan (2023) |
| *Synanthedon myopaeformis* | 15,176 | 78.68 | 3,697 | 76.29 | 762 | 85.43 | 1,289 | 84.02 | 1,439 | 82.28 | 401 | 95.51 | OX122944^c^ | Langdon and Holland (2024) |
| ***Synanthedon namdoelegans*** | **15,578** | **79.18** | **3,698** | **76.50** | **776** | **85.82** | **1,282** | **85.10** | **1,457** | **83.46** | **723** | **91.29** | **PV762247** | **This study** |
| *Synanthedon vespiformis* | 17,252 | 79.76 | 3,698 | 75.08 | 761 | 85.15 | 1,289 | 83.86 | 1,437 | 82.32 | 2,458 | 94.51 | OU906976^c^ | Boyes and Lees (2022) |
| Average | 15,654 | 79.46 | 3,707 | 76.99 | 771 | 85.27 | 1,321 | 83.72 | 1,464 | 81.79 | 722 | 94.52 |  |  |

^a^For protein-coding genes (PCGs), termination codons were excluded from the total codon count. ^b^Partially re-annotated for the target region. ^c^The whole mitochondrial genome was annotated in this study.
